# Immunogenicity Analysis and Identification of Potential T-Cell Epitopes in C129R Protein of African Swine Fever Virus

**DOI:** 10.3390/microorganisms12061056

**Published:** 2024-05-24

**Authors:** Wenzhu Zhai, Ying Huang, Yuheng He, Yuanyuan Chu, Chunhao Tao, Zhongbao Pang, Zhen Wang, Hongfei Zhu, Hong Jia

**Affiliations:** Institute of Animal Sciences, Chinese Academy of Agricultural Sciences, Beijing 100080, China; shandixhqm@163.com (W.Z.); hy811cysbm@163.com (Y.H.); heyuheng2012@163.com (Y.H.); 18522961671@163.com (Y.C.); chunhao_tao@163.com (C.T.); 82101205453@caas.cn (Z.P.); wz2893963594@126.com (Z.W.); bioclub@vip.sina.com.cn (H.Z.)

**Keywords:** African swine fever virus, C129R protein, humoral and cellular immunity, T-cell epitope

## Abstract

The highly conserved C129R protein of AFSV was utilized in the development of an ASFV recombinant adenovirus vaccine, demonstrating strong immunogenicity. In this study, we immunized 6-week-old female C57BL/6J mice via subcutaneous injection with 10 μg of purified C129R protein. Humoral and cellular immune effects were assessed using ELISA, flow cytometry, and ELISpot assays. Additionally, 19 peptides of the C129R protein were synthesized and screened for the use of bioinformatics. Positive T-cell epitopes were screened using ELISpot. The results indicated a higher proportion of CD4+ and CD8+ T lymphocytes in immunized mice compared to control mice. ELISA analysis revealed a serum titer of approximately 1:1, 638, 400 in the experimental group of mice. Additionally, peptides C11^(53−61aa)^, C14^(81−89aa)^, C16^(97−105aa)^, and C18^(116−124aa)^ from the C129R protein were able to activate mice spleen lymphocytes to produce IFN-γ. These findings suggest that the C129R protein significantly enhances both humoral and cellular immunity in immunized mice. Moreover, peptides C11, C14, C16, and C18 may serve as potential T-cell epitopes for the C129R protein. These results lay the groundwork for the further exploration of ASFV C129R protein and the identification of novel ASF vaccine antigens.

## 1. Introduction

African Fever Swine (ASF) was first reported in Kenya in 1921 and has spread to 35 African countries. Contact with wart pigs is the main cause for outbreaks [1,2]. In 2018, the first ASF outbreak was confirmed in Shenyang, Liaoning Province, China. Due to the small-scale, low biosecurity level of pig farms in China, ASF rapidly spread throughout China, threatening the pig-farming industry [3,4,5]. African swine fever virus (ASFV) is a linear double-stranded DNA virus with an icosahedral symmetric structure, consisting of a nucleoid, matrix, membrane, capsid, and outer envelope. Its genome is 170–194 kb, with over 150 open reading frames (ORFs), and approximately 200 proteins, many of which are functional in viral replication, immune escape, and transmission [6].

The increasing spread of ASF highlights the urgent need for a safe and effective vaccine [7]. At present, the bottleneck for existing ASF subunit vaccines, viral live vector vaccines, and mRNA vaccines is that none of them have known viral antigens that induce complete protection [8,9]. Studies indicate that neutralizing antibodies are insufficient to provide adequate protection [10,11], emphasizing the importance of CD8+ T-cell-mediated immunity for ASF protection [9]. T-cells recognize short peptides presented by Antigen-Presenting Cells (APC) via the Major Histocompatibility Complex (MHC), underscoring the importance of screening for ASFV proteins and the use of effective T-cell epitopes to activate T-cell immune responses.

Immunization with C129R recombinant adenovirus can induce cellular and humoral immunity in pigs [11,12]. However, research on T-cell epitopes of the C129R protein is limited. In this study, we administered the C129R protein subcutaneously to C57BL/6J mice. Subsequently, 7 days post secondary immunization, we conducted an evaluation of both cellular and humoral immune responses using ELISA, flow cytometry, and ELISpot assays. The C129R protein T-cell epitope was predicted using the IEDB server V2.22 and NetMHCpan-4.1 server, with screening being conducted using the ELISpot method. This study will elucidate the immunogenicity of C129R, aid in the development of effective ASF vaccines, and serve as a technical reference for identifying ASFV antigens and T-cell epitopes.

## 2. Materials and Methods

### 2.1. Cell and Protein

The HEK293F cell line was obtained from Chinese Academy of Sciences Cell Bank and maintained in our laboratory under standard conditions. HEK293F cells were cultured in Expi293^TM^ Expression Medium (Gibco, Grand Island, NE, USA, Cat. No#.A14351-01). Mouse splenic lymphocytes were prepared and cultured in RPMI 1640 medium (Gibco, Grand Island, NE, USA, Cat. No#. 11875093) supplemented with 10% fetal bovine serum (FBS) (Gibco, Grand Island, NE, USA, Cat. No#. A31604-01) and 1% penicillin–streptomycin (Gibco, Grand Island, NE, USA, Cat. No#. 15140-122).

### 2.2. Animals

Specific pathogen-free (SPF)-grade, 6-week-old, female C57BL/6J mice were purchased from Beijing Viton Lihua Laboratory Animal Technology Co., Ltd. (Beijing, China). and were raised in a negative-pressure barrier environment (partially isolated environment) at the Animal Experiment Center of the Beijing Institute of Animal Husbandry and Veterinary Medicine, Chinese Academy of Agricultural Sciences. Animal experiments can be conducted at the second level of biosafety (ABSL-2).

### 2.3. Expression and Purification of Recombinant C129R Protein

HEK293F cells were inoculated into a 250 mL cell culture shaker and incubated at 37 °C, 120 rpm, in the shaker incubator with 5% CO_2_. The eukaryotic expression plasmid pMAL-Fc-C129R was transfected into 100 mL of 293F cells using Polyethylenimine Linear (PEI) MW40000 (Yeasen, Shanghai, China, Cat. No #. 40816ES02), and the growth status and rate of the cells were examined within 5 days of transfection. The supernatant of the cells was collected aseptically on the 5th day of transfection for the detection of the target protein. The collected supernatant was subjected to gel affinity chromatography with AKTA purifier UPC10 using Protein A resin. The purified target proteins were analyzed by Coomassie brilliant blue staining, and then the solution was replaced and concentration was determined. Finally, the concentration of the final protein was determined by BCA kit (Thermo Fisher, Grand Island, NE, USA, Cat. No#. A55864) and the target proteins were stored in the refrigerator at −80 °C.

### 2.4. Western Blot Analysis

The proteins were mixed with 5× loading buffer at 100 °C for 10 min. Proteins were separated by SDS-PAGE using 4–20% SurePAGE preformed gel (Genscript, Nanjing, China, Cat. No #. M42012C), and the proteins were transferred onto the NC film from the SDS-PAGE using the wet method (80 V, 30 min; 120 V, 50 min). The NC film was blocked with 5% skimmed milk powder sealing solution and was gently shaken at room temperature for sealing for 2 h. Then, the NC film was washed 5 times with TBST. The positive pig serum diluted with the first antibody diluent was incubated at 4 °C overnight, and TBST was cleaned 5 times. The second antibody was horseradish peroxide (HRP)-labelled goat anti-mice antibody (Zsbio, Beijing, China, Cat. No # ZF-0513) diluted with 5% skimmed milk powder and incubated at room temperature for 1 h. After washing the film, the blots were displayed using Tanon™ Femto-sig ECL Western Blotting Substrate (Tanon. Shanghai, China, Cat. No#. 180-506) and analyzed using BIO-RAD gel imager (Shanghai, China).

### 2.5. Screen of the T-Cell Epitopes on the C129R Protein

The T-cell epitope region of the C129R protein was predicted by IEDB server V2.22 (https://www.iedb.org/, accessed on 4 September 2023) and the NetMHCpan-4.1 server (https://services.healthtech.dtu.dk/services/NetMHCpan-4.1, accessed on 4 September 2023). The SLA-1*0401, SLA-2*0401, and SLA-3*0401 alleles were used to generate epitopes. The parameters used for the analysis were as follows: the peptide length was 9aa and percentile rank was 1. The peptides were screened according to the percentile rank and secondary structural features, and the lower the rank% value, the higher the peptide affinity. Based on these comprehensive results, 19 high-affinity peptides were identified and numbered from C1 to C19 (Table 1). These peptides covered the entire length of the C129R protein. The peptides were synthesized by GenScript Biotech Co., Ltd. (Nanjing, China). The purity of the peptides was greater than 95%, and each peptide was dissolved in ddH_2_O, DMSO or formic acid (CH_2_O_2_), as recommended by the COA report.

### 2.6. Animal Immunization

**Figure 1:**
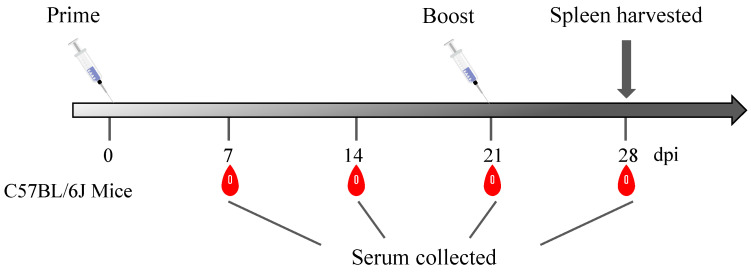
Schematic diagram of the immunization and sample collection. Black arrow shows that mouse spleens were collected at 28 dpi.

C57BL/6J mice were randomly allocated to two distinct groups, labeled Group A and Group B, with six mice in each group. Group A was immunized with 10 μg of C129R protein, and Group B was immunized with 0.2 mL of PBS as a negative control. Before immunization, the proteins were diluted in sterile PBS. All mice were immunized with the same dose at 21 days post immunization (dpi). Blood serum samples were obtained at seven-day intervals after vaccine administration. The lymphocytes were isolated from the spleen at 28 dpi (Figure 1).

### 2.7. Isolation Mice Spleen Lymphocytes

Mouse splenic lymphocytes were isolated using a mouse splenic lymphocyte isolation kit (Solarbio, Beijing, China, Cat. No#. P8860). On the 7th day of secondary immunization, six pieces of mouse were taken from each group, immersed in 75% ethanol for 10 min after euthanasia, and then separated on an ultra-clean bench. A total of 5 mL of mouse lymphocyte diluent was aspirated with a syringe, the needle was lodged in the spleen end, and the cells were blown out onto a 35 mm petri dish placed with a 200-mesh membrane; the spleens were then placed on the membrane and the cell suspension was obtained by gently crushing the spleen with a syringe. According to the instructions of the mouse splenic lymphocyte isolation kit, the diluted splenic cell suspension was placed into a 15 mL centrifuge tube containing 5 mL of the isolation medium and centrifuged slowly at 800× *g* for 30 min at room temperature. The intermediate lymphocyte layer was aspirated and washed twice with PBS. Finally, the mouse splenic lymphocytes were resuspended in RPMI 1640 complete culture medium.

### 2.8. IFN-γ ELISpot Assays

**Protein.** Antigen-specific IFN-γ-secreting cells were quantified by ELISpot assays using the mouse IFN-γ precoated ELISpot kit (Dakewe, Shenzhen, China, Cat. No#2210006) and the cell concentration was adjusted to 2 × 10^6^ cells/mL according to the manufacturer’s protocol. A total of 100 μL of cells/well were inoculated into 96-well plates, and each well was stimulated with 10 μL of C129R recombinant protein (10 μg/mL) to stimulate lymphocytes in vitro. PMA and ionomycin were included as positive controls, and no stimulant was added as a negative control. After being stimulated at 37 °C in an incubator with 5% CO_2_ for 24 h, the stimulants were discarded. The cells were lysed with pre-cooled ultrapure water for 10 min, and then biotinylated antibody was added and incubated for 1 h at 37 °C. Then, the plate was washed 6 times with washing buffer, and streptavidin–HRP was added and incubated for 1 h at 37 °C. Following another six washes with washing buffer, AEC colorant was added and incubated for 30 min at 37 °C. After a final wash with ultrapure water, the plates were air-dried in a cool, ventilated area. After the plates were completely dry, spot-forming units (SFU) were counted on an IRIS Mabtech ELISpot plate reader. In this study, the number of SFUs was determined using the following rule: if the results of the PMA-positive control wells were normal, the SFUs of the test wells minus the SFUs of the cellular control wells would be the number of specific SFUs produced by protein stimulation.

**Peptide.** The experimental wells were stimulated with 10 μL of peptide (100 μg/mL), positive wells were stimulated with PMA and Ionomycin, negative control wells received no stimulants, and the wells were incubated for 20 h [13]. The remaining steps were the same as those for the protein ELISpot.

### 2.9. Indirect ELISA

An indirect enzyme-linked immunosorbent assay (ELISA) was used to analyze the serum of each group of mice. The ELISA plate was incubated with 100 μL C129R recombinant protein (2 μg/mL) overnight at 4 °C. Before coating the plates, the proteins were diluted with 50 mM phosphate buffer (pH 9.5). The plates were washed 3 times with PBST and closed by adding skimmed milk for 2 h at 37 °C. The diluted mouse serum (dilution of the first well 1:3200) was diluted, added to the plates, and incubated for 1 h at 37 °C. Negative serum from PBS-immunized mice was used as a control. After washing three times with PBST, 100 μL of horseradish-labeled goat anti-mice IgG antibody (1:5000 dilution) (Abcam, Cambridge, UK, Cat. No#ab6789) was added as the secondary antibody and incubated for 1 h at 37 °C. Following another three washing steps, 100 μL of substrate TMB color development solution (KPL, Milford, MA, USA, Cat. No#5120-0077) was added and incubated for 15 min at 37 °C in the dark. A total of 50 μL of a 1M HCl termination solution was used to stop the reaction. The optical density (OD) of each well was measured at 450 nm using a microplate reader (Multiskan GO, Grand Island, NE, USA). An OD450 nm value of experimental wells/blank wells (P/N) ≥ 2.1 was judged as positive, and the OD450 nm of P/N value < 2.1 was judged as negative.

### 2.10. CD4+/CD8+ T-Cell

Flow cytometry was used to detect lymphocyte subtypes. A total of 100 μL of single-cell suspension prepared in RPMI 1640 complete medium was added to the centrifuge tube, centrifuged at 200× *g* for 5 min, and the supernatant was discarded. Cells were resuspended in 97 μL of cell staining buffer (BioLegend, San Diego, CA, USA, Cat. No#420201). Cells were first stained with 0.5 μL of anti-mice CD16/32 antibody (0.5 mg/mL) (BioLegend, San Diego, CA, USA, Cat. NO# 156604) to block the nonspecific binding of immunoglobulins to Fc receptors, and then stained with the following fluorescently labeled antibodies: 0.5 μL of FITC anti-mice CD3ε antibody (0.5 mg/mL) (BioLegend, San Diego, CA, USA, Cat. NO# 100204), 1.25 μL of PerCP/Cyanine5.5 anti-mice CD8α antibody (0.2 mg/mL) (BioLegend, San Diego, CA, USA, Cat. NO# 100734) and 1.25 μL PE anti-mice CD4 antibody (0.2 mg/mL) (BioLegend, San Diego, CA, USA, Cat. NO# 100512). After incubation for 30 min on ice, the mixture was centrifuged at 200× *g* for 5 min, the supernatant was discarded, and two washes with PBS were performed. Finally, the cells were resuspended in 250 μL of Cell Staining Buffer per flow tube, CD4+ T-cells and CD8+ T-cells were acquired using BD FACSVerse cell analyzer (BD Bioscience, Franklin Lakes, NJ, USA), and data were analyzed using FlowJo software V_10.

### 2.11. Detection of IFN-γ, IL-2, IL-4, and IL-10

For the evaluation of cellular and humoral immunity, the production of IFN-γ, IL-2, IL-4, and IL-10 in mouse serum at 21 and 28 dpi was measured using a Q-Plex™ Mouse HS kit (Cayman, Michigan, USA, Cat, No#39970), which was used according to the manufacturer’s instructions.

### 2.12. Conservative Analysis of the T-Cell Epitopes

To analyze the conservation of the T-cell epitopes screened in this experiment, Table 2 shows 16 representative ASFV strains of all genotypes. The corresponding amino acid sequences of C129R were aligned using MEGA 11.

### 2.13. Statistical Analysis

Statistical analyses and plots were analyzed using GraphPad Prism 9.0 software (GraphPad Software Inc., San Diego, CA, USA). The normality of data distribution was assessed using the Shapiro–Wilk test for each dataset, and an unpaired *t*-test was used to determine whether the differences between groups were statistically significant (* *p* < 0.05, ** *p* < 0.01, *** *p* < 0.001).

## 3. Results

### 3.1. C129R Protein Was Expressed in 293F

**Figure 2:**
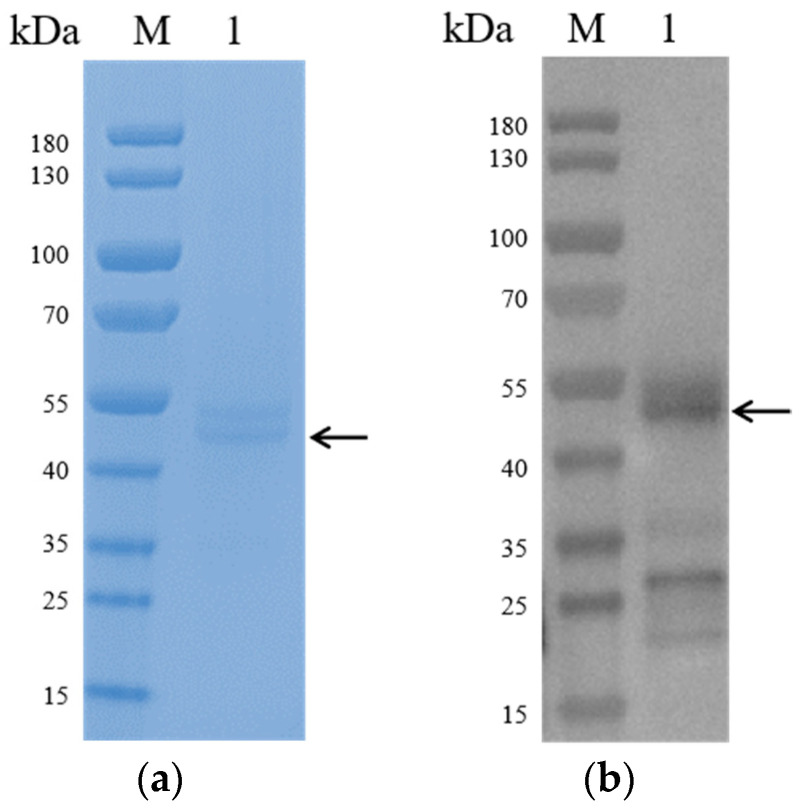
To verify the expression of C129R, pMAL-Fc-C129R was transfected into 293F cells, and the supernatant was collected after five days of transfection. (**a**) SDS-PAGE confirmed the Fc-tagged recombinant protein obtained by affinity purification; the molecular weight of the bands was about 48 kDa. M is a protein ladder. Arrows represent target proteins. (**b**) Western blot analysis of C129R protein with anti-ASFV antibody serum. Arrows represent target proteins.

ASFV C129R recombinant protein was purified by affinity column, and the expression of ASFV C129R in 293F was detected by immunoblotting. The SDS-PAGE results showed that a clear target band was detected at 48 kDa (Figure 2a), which was consistent with the expected size and indicated that the ASFV C129R recombinant protein was correctly expressed. The purified recombinant protein was verified and showed good reactivities with anti-ASFV antibody serum (Figure 2b).

### 3.2. C129R Protein Induced the Humoral Immune Response

**Figure 3:**
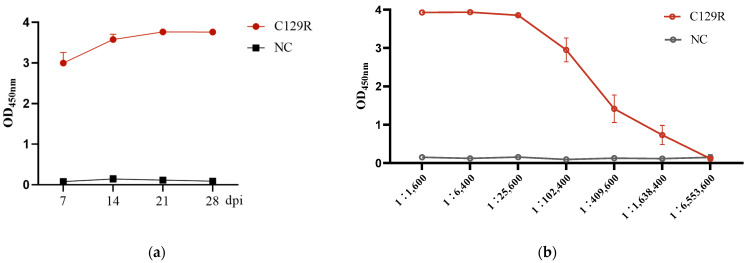
Detection of antibody levels to C129R protein in the serum of immunized mice. C129R-specific IgG antibodies were plotted against sera on different days of immunization. (**a**) Results of the serum antibody levels. (**b**) Antibody titers at the 7th day post second immunization.

Blood was collected from the orbital sinuses of immunized C57BL/6J mice, and serum antibody levels against the C129R protein were detected with ELISA. The findings indicate that, unlike Group B, Group A exhibited detectable antibodies to the C129R protein on the 7th day post first immunization, with antibody titers reaching 1:1, 638, 400 on the 7th day post second immunization. The antibody levels increased 2–3 weeks after the first immunization, peaked on the 21 dpi, and then stabilized (Figure 3).

### 3.3. C129R Protein Activated the Cellular Immunity and Induced IFN-γ Production

**Figure 4:**
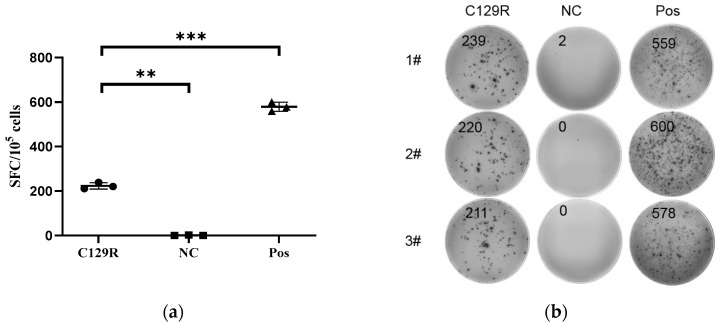
IFN-γ response of splenic lymphocytes from mice immunized with C129R protein. Splenic lymphocytes from mice vaccinated with the C129R protein were collected and stimulated with C129R protein for in vitro restimulation for IFN-γ ELISpot. (**a**) Results of the assay for IFN-γ in immunized mice are presented as SFC/10^5^ cells, with the mean stimulated spots in each group presented as a bar graph and the error bars representing the standard deviation. Statistically significant differences are indicated by asterisks. ** *p* < 0.01; *** *p* < 0.001. (**b**) ELISpot Spot Plot. 1#–3# indicates 3 representative mice.

Seven days after the booster immunization, splenic lymphocytes from the mice were isolated and assessed for the cellular immunity response activated by the C129R protein using the mouse IFN-γ precoated ELISpot kit (Dakewe, Cat. No#2210006). The results (Figure 4a,b) indicated a notable increase in the number of IFN-γ-producing T-cells in mice immunized with the C129R protein (Group A) compared to the control groups (Group B). This suggests that the C129R protein effectively triggers cellular immunity and could serve as a target for the further screening of ASFV T-cell epitopes.

### 3.4. C129R Protein Increased CD4+/CD8+ T-Cell Ratios of Splenic Lymphocytes

**Figure 5:**
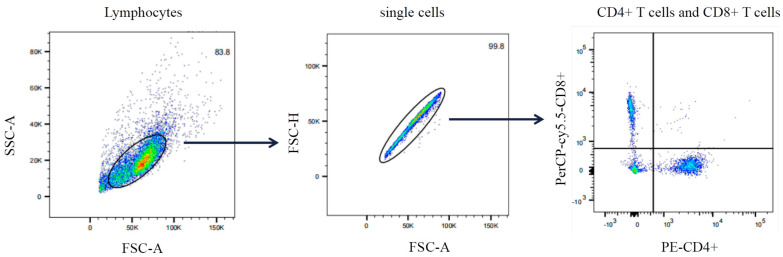
Gating strategy for lymphocyte staining flow cytometry. A gate was drawn on the FSC and SSC plot to circle lymphocytes, excluding fragments and double cells. A scatter plot was created with CD4+ and CD8+ T-cells’ expression levels as the axes and different gates were set to distinguish between CD4+ and CD8+ T-cells.

**Figure 6:**
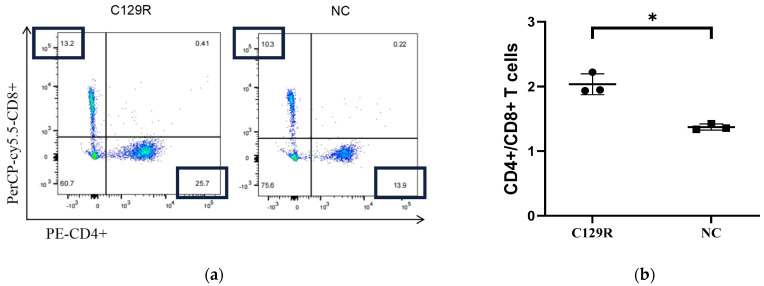
T-cell subpopulation test results. (**a**) The representative flow cytometry plots are presented. (**b**) CD4+/CD8+ T-cell ratios were analyzed by flow cytometry. The CD4+/CD8+ T-cell ratios of the experimental group (Group A) and the blank group (Group B) were 1.946 and 1.35. Statistically notable variations in components are denoted by asterisks. * *p* < 0.05.

The effect of the C129R protein on the ratio of different T-cell subpopulations was detected by flow cytometry. The circle-gate strategy for streaming is illustrated in Figure 5. According to the results (Figure 6a,b), the C129R protein-immunized groups showed an increase in CD4+ and CD8+ T-cells, and a significant difference in the CD4+/CD8+ T-cell ratio (*p* < 0.05), indicating that the C129R protein induced humoral and cellular immunity responses in the mice.

### 3.5. Effect of C129R Protein on Th1 and Th2 Immune Responses

**Figure 7:**
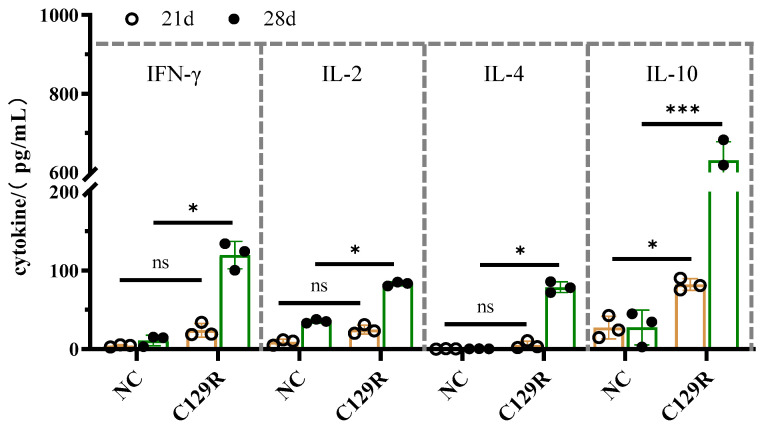
Cytokines detection results. IFN-γ, IL-2, IL-4, and IL-10 detection by ELISA in mouse serum collected on days 21 and 28 after the first immunization with the C129R protein. Statistically notable variations in each group are denoted by asterisks. ns, *p* > 0.05; * *p* < 0.05; *** *p* < 0.001.

To determine the levels of IFN-γ, IL-2, IL-4, and IL-10 using ELISA (Figure 7), serum was collected from immunized mice. Compared to the control group, the C129R protein immunization increased the expression of Th1-type cytokine (IFN-γ and IL-2) and Th2-type cytokine (IL-4 and IL-10). The cytokine content at the two time points before and after the strengthening immunization was higher than that in the control group (*p* < 0.05). These results suggest that the C129R protein, after the first strengthened immunization, could stimulate the mouse to secrete immune-related cytokines in large quantities and elevate strong immune responses.

### 3.6. C11^(53−61aa)^, C14^(81−89aa)^, C16^(97−105aa)^, and C18^(116−124aa)^ Were the Potential T-Cell Epitopes in C129R Protein

**Figure 8:**
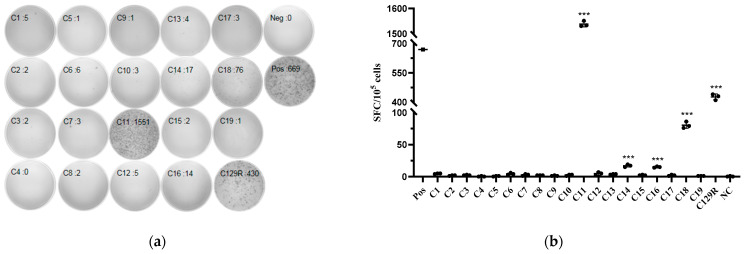
Four T-cell epitopes were identified on the C129R protein. IFN-γ responses were detected using ELISpot in the splenic lymphocytes of mice inoculated with C129R protein. (**a**) ELISpot spot graph (only one representative result is shown). (**b**) ELISpot bar chart. IFN-γ responses were detected in the splenic lymphocytes of mice inoculated with C129R protein using ELISpot. *** *p* < 0.001.

To further screen for ASFV-specific T-cell epitopes, 19 predicted 9aa-length T-cell epitopes were predicted and synthesized. The ELISpot results, obtained 7 days after secondary immunization with C129R protein, revealed differential IFN-γ secretion levels by splenic lymphocytes from the two groups under three stimulation conditions (peptide, protein, and positive stimulant). The SFUs in the cellular control wells without peptide stimulation were 0. Subtracting 0 from the SFUs of the test wells will yield the cell number of specific SFUs produced by the peptide stimulation. Therefore, as shown in Figure 8, peptide C11 (^53^LQNPYEAVI^61^), peptide C14 (^81^GHVTWAVPY^89^), peptide C16 (^97^AKPDAIMLT^105^), and peptide C18 (^116^ALNQNVLTL^124^) had strong specificity for IFN-γ production.

### 3.7. Epitope Conservation Analysis

**Figure 9:**
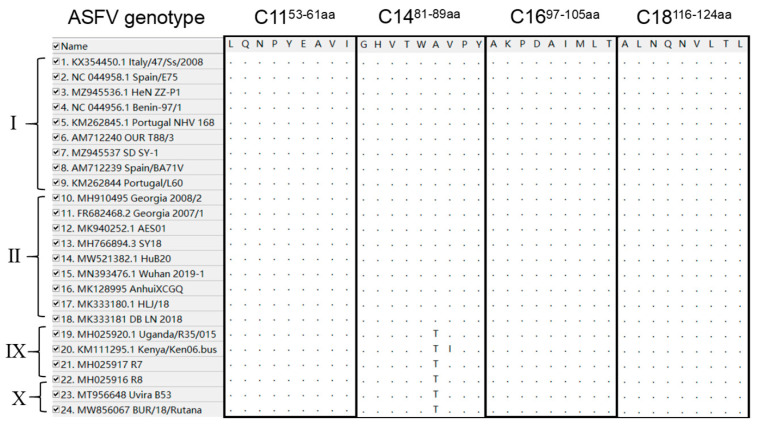
Sequence conservation analysis of four identified epitopes among twenty-four ASFV reference strains collected from NCBI using MEGA 11 Multiple Sequence Comparison. The sequences of the four epitopes were surrounded by black frames.

A conservation analysis of the four epitopes, C11, C14, C16, and C18, among different ASFV genotypes was performed using MEGA 11. As shown in Figure 9, epitopes C11, C16, and C18 were highly conserved among all the analyzed ASFV genotype strains. Epitopes C14 were conserved among the analyzed ASFV genotype I and II strains.

## 4. Discussion

The prevention and control of African swine fever (ASF) presents multifaceted challenges. Although inactivated ASF vaccines can elicit a humoral immune response characterized by the production of specific antibodies, they do not confer effective immune protection [14]. An attenuated ASFV vaccine, created by knocking out virulence genes in live virus particles, replicates in the host and mimics natural infections to trigger humoral and cellular immunity. However, the simultaneous deletion of multiple virulence genes does not guarantee the absence of virulence, nor does it reduce the risk of persistent infection or ensure the induction of a protective immune response [15,16]. In contrast, the subunit vaccines offer distinct safety advantages. The key to developing effective subunit vaccines lies in the identification of the optimal combination of protective antigens that maximally stimulate the immune system, along with the selection of adjuvants that enhance antigen delivery [17]. The ability to induce neutralizing antibodies and activate cytotoxic T-lymphocyte responses significantly contributes to resistance against ASFV infection [18,19]. In this experiment, mice were immunized with the expressed C129R protein, resulting in activation of ASFV-specific humoral and cellular immune responses.

Numerous studies have highlighted the importance of cellular immune responses in defending against ASFV, extending beyond humoral immune responses that produce neutralizing antibodies. These cellular responses, particularly those involving cytotoxic T lymphocytes and natural killer (NK) cells, are crucial, with interferon-gamma (IFN-γ) serving as a significant biomarker for assessing cellular immune activation [3,20]. A DNA vaccine was designed to encode a fusion protein consisting of p30 and p54. This vaccine successfully induced a notable cytotoxic T-lymphocyte (CTL) response. However, it did not elicit a significant antibody response. This CTL response conferred a degree of protection against homologous ASFV virus attacks [21].

T-cell epitopes bind to MHC molecules to induce immune responses and the primary requirement for T-cell recognition is binding to MHC; the prediction of antigenic peptide-MHC affinity is a key issue in T-cell epitope screening. Protein antigens must be processed and presented as antigenic peptides by APCs to bind to major histocompatibility complex (MHC) I and MHC II molecules [22,23], and T-cell epitope-synthesizing peptides bind directly to MHC molecules [24]. T-cell epitopes were screened using three strategies: the computer prediction of synthetic peptides, immunopeptidomics’ identification of peptides, and the transfection of fibroblasts encoding the full-length protein granule of ASFV. Remarkably, all three methods identified the same CD8+ T-cell epitopes [25]. Netherton et al. [11] predicted peptides corresponding to 133 proteins encoded by ASFV OUR T88/3, screened 18 viral proteins identifiable by ASFV-immunized porcine PBMCs, and experimentally determined specific antigenic epitopes. A multi-epitope vaccine comprising B- and T-cell epitopes from ASFV p12, p17, p22, p54, p72, and CD2v proteins elicited immune responses in immune simulations [26]. Zhang et al. [27] constructed two fusion proteins, ZPM (p30-modified p54-Z12) and ZPMT (p30-modified p54 T-cell epitope). Immunization experiments in mice revealed that ZPMT, containing T-cell epitopes, induced stronger cellular immunity and exhibited a superior ASFV neutralization capacity. In this study, 19 T-cell antigenic epitopes of the ASFV C129R protein were predicted using the IEDB server V2.22 and NetMHCpan-4.1. Four epitope peptides, namely C11, C14, C16, and C18, capable of activating ASFV-specific splenic lymphocytes to produce IFN-γ, were identified through ELISpot screening. These findings enhance our understanding of T-cell epitope dynamics and provide insights for the development of targeted vaccines aimed at eliciting a comprehensive immune response against ASFV.

ASFV utilizes its C129R protein to target and degrade intracellular cyclic GMP-AMP (2′,3′-cGAMP), thereby inhibiting the cGAS-STING signaling pathway and evading the host’s antiviral immune response [28]. An epitope denotes a specific region of a protein recognized by the immune system and may not encompass the complete structural domains required for specific biological functions. Hence, expressing only a portion of the C129R protein epitope might not completely mimic the immunosuppressive functions of the entire protein. However, this relies on whether the epitopes encompass the crucial functional regions linked to immunosuppression. If the epitopes contain these pivotal regions, they might exert immunosuppressive activity. Therefore, it was essential to confirm whether the epitopes identified in this study were indeed key functional regions for the C129R protein to exert its immunosuppressive effects.

In conclusion, we characterized the immunogenicity of the ASFV C129R protein, conducted a comprehensive screening of T-cell epitopes of the C129R protein, and identified four T-cell epitopes for the first time, which were confirmed to be effective in stimulating the secretion of IFN-γ through the ELISpot results. The epitope C11^(53−61aa)^ demonstrated a remarkable ability to activate lymphocytes and elicit enhanced T-cell responses compared to those induced by the full C129R protein. Particularly, the C11 epitope showed significant promise for further investigation and potential inclusion in an epitope-based vaccine. However, comprehensive functional validation and in-depth studies in pig herds with diverse SLA alleles are necessary to fully assess the immunogenicity of these T-cell epitopes.

## 5. Conclusions

In our study, we found that the C129R protein stimulated both humoral and cellular immune responses. We identified four immunodominant epitopes within the C129R protein: C11 (^53^LQNPYEAVI^61^), C14 (^81^GHVTWAVPY^89^), C16 (^97^AKPDAIMLT^105^), and C18 (^116^ALNQNVLTL^124^). These epitopes are highly conserved across ASFV genotype I and II strains and can trigger cellular immune responses. Our results lay a robust foundation for further exploration of the antigenic role of ASFV C129R and the development of effective ASF subunit vaccines, viral live vector vaccines, and mRNA vaccines.

## Figures and Tables

**Table 1:** Nineteen high-affinity peptides of C129R protein.

Number	Start–End	Peptide	Solvent	Number	Start–End	Peptide	Solvent
C1	1–9	MEHPSTNYT	ddH_2_O	C2	6–14	TNYTPEQQH	ddH_2_O
C3	12–20	QQHEKLKHY	ddH_2_O	C4	18–26	KHYVLIPKH	ddH_2_O
C5	20–28	YVLIPKHLW	DMSO	C6	25–33	KHLWSYIKY	DMSO
C7	31–39	IKYGTHVRY	ddH_2_O	C8	38–46	RYYTTQNVF	DMSO
C9	43–51	QNVFRVGGF	ddH_2_O	C10	48–56	VGGFVLQNP	ddH_2_O
C11	53–61	LQNPYEAVI	ddH_2_O	C12	58–63	EAVIKNEVK	CH_2_O_2_
C13	77–85	TKAKGHVTW	ddH_2_O	C14	81–89	GHVTWAVPY	ddH_2_O
C15	88–96	PYDNISKLY	DMSO	C16	97–105	AKPDAIMLT	ddH_2_O
C17	109–117	NVEKALHAL	ddH_2_O	C18	116–124	ALNQNVLTL	DMSO
C19	121–129	VLTLASKIR	DMSO				

**Table 2:** ASFV isolates and GenBank accession numbers.

Genotype	Strain	GenBank Accession No.	Source
I	Portugal/L60	KM262844	Portugal
	SD SY-1	MZ945537	China
	HeN ZZ-P1	MZ945536.1	China
	Italy/47/Ss/2008	KX354450.1	Italy
	OUR T88/3	AM712240	Portugal
	Spain/E75	NC_044958.1	Spain
	Spain/BA71V	AM712239	Spain
	Benin-97/1	NC_044956.1	Benin
	Portugal/NHV/1968	KM262845.1	Portugal
II	Georgia_2007/1	FR682468.2	Georgia
	Georgia_2008/2	MH910495	Georgia
	AES01	MK940252.1	China
	HuB20	MW521382.1	China
	SY18	MH766894.3	China
	Wuhan 2019-1	MN393476.1	China
	AnhuiXCGQ	MK128995	China
	HLJ/18	MK333180.1	China
	DB/LN/2018	MK333181.1	China
IX	R7	MH025917	Uganda
	R8	MH025916	Uganda
	Kenya/Ken06.Bus	KM111295.1	Kenya
	Uganda/R35/2015	MH025920.1	Uganda
X	Uvriva B53	MT956648	Democratic Republic of the Congo
	BUR/18/Rutana	MW856067	Burundi

## Data Availability

The original contributions presented in the study are included in the article, further inquiries can be directed to the corresponding author.
